# Spatial and temporal variability in ringed seal (*Pusa hispida*) stable isotopes in the Beaufort Sea

**DOI:** 10.1002/ece3.6186

**Published:** 2020-03-24

**Authors:** Nicole P. Boucher, Andrew E. Derocher, Evan S. Richardson

**Affiliations:** ^1^ Department of Biological Sciences University of Alberta Edmonton AB Canada; ^2^ Wildlife Research Division, Science and Technology Branch Environment and Climate Change Canada Winnipeg MB Canada

**Keywords:** Beaufort Sea, climate change, niche width, *Pusa hispida*, ringed seal, stable isotopes

## Abstract

Arctic ecosystem dynamics are shifting in response to warming temperatures and sea ice loss. Such ecosystems may be monitored by examining the diet of upper trophic level species, which varies with prey availability. To assess interannual variation in the Beaufort Sea ecosystem, we examined spatial and temporal trends in ringed seal (*Pusa hispida*) δ^13^C and δ^15^N in claw growth layers grown from 1964 to 2011. Stable isotopes were correlated with climate indices, environmental conditions, seal population productivity, and geographic location. Sex and age did not influence stable isotopes. Enriched ^13^C was linked to cyclonic circulation regimes, seal productivity, and westward sampling locations. Higher δ^15^N was linked to lower sea surface temperatures, a higher percentage of pups in the subsistence harvest, and sample locations that were eastward and further from shore. From the 1960s to 2000s, ringed seal niche width expanded, suggesting a diversification of diet due to expansion of prey and/or seal space use. Overall, trends in ringed seal stable isotopes indicate changes within the Beaufort Sea ecosystem affected by water temperatures and circulation regimes. We suggest that continued monitoring of upper trophic level species will yield insights into changing ecosystem structure with climate change.

## Graphical abstract

To assess interannual variation in the Beaufort Sea ecosystem, we examined spatial and temporal trends in ringed seal (*Pusa hispida*) δ13C and δ15N in claw growth bands grown between 1964 and 2011. Stable isotopes correlated to climate indices, environmental conditions, seal population productivity, and geographic location. From the 1960s to 2000s, ringed seal niche width expanded, suggesting a diversification of diet due to expansion of prey and/or seal space use.

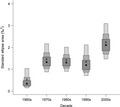

## 1 INTRODUCTION

Monitoring of Arctic marine ecosystems can be challenging, due to the region's remoteness, large scale, and complexity. However, identifying and assessing shifts in ecosystem dynamics will aid management and conservation efforts, particularly as the Arctic undergoes large‐scale reductions in sea ice due to climate change (Galley et al., 2016; Holland, Bitz, & Tremblay, 2006; Parkinson, 2014; Stroeve, Markus, Boisvert, Miller, & Barrett, 2014). Arctic sea ice extent in September declined at a rate of 9.1%/decade between 1979 and 2006 (Stroeve, Holland, Meier, Scambos, & Serreze, 2007), with predictions for continued sea ice declines and a possible ice‐free September by the end of the 21st century (Overland & Wang, 2013; Wang & Overland, 2009). From 1979 to 2013, Arctic sea surface temperatures rose by 0.5–1.5°C, and the melt season increased 5 days/decade (Stroeve et al., 2014). These shifting environmental conditions, among others, will likely result in changes to the distribution and abundance of species, which in turn alters community composition (Carroll, Horstmann‐Dehn, & Norcross, 2013; Grebmeier, Overland, et al., 2006; Montevecchi & Myers, 1996; Rose, 2005; Young & Ferguson, 2014). Changes in ecosystem dynamics may be monitored by tracking the stable isotopes of upper trophic level, generalist species, under the assumption that the composition of their diets reflects prey availability and that prey isotopic values are distinct (Braune, Gaston, Hobson, Gilchrist, & Mallory, 2014; McKinney et al., 2013; Yurkowski, Ferguson, Semeniuk, et al., 2016).

Carbon (δ^13^C) and nitrogen (δ^15^N) stable isotopes from assimilated resources are integrated over time into the consumer's tissues, creating a record of their diet (Ben‐David & Flaherty, 2012). Integration of stable isotopes into tissues is based on tissue‐specific metabolisms; therefore, tissues with higher metabolic activity will represent a more recent diet (Carleton, Kelly, Anderson‐Sprecher, & Rio, 2008; Hobson & Clark, 1992). For metabolically inert tissues (e.g., claw, hair), stable isotopes are incorporated during growth and retained following cessation of metabolic activity (Hobson & Clark, 1992; Rubenstein & Hobson, 2004). Further, a predictable shift in stable isotopes occurs between the food source and consumer tissues during digestion and assimilation, termed trophic discrimination (Peterson & Fry, 1987). For example, nitrogen typically enriches approximately 3.8‰ per trophic level (Hobson & Welch, 1992). Therefore, a consumer's tissues will have a mean isotopic value based on food consumed, which is influenced by the tissue's turnover rate, trophic discrimination, and other environmental factors (e.g., regional isotope baselines; Dalerum & Angerbjorn, 2005; McCutchan, Lewis, Kendall, & McGrath, 2003). Stable isotopes may be used to infer niche width of an animal, which indicates breadth of isotopically distinct prey sources eaten (Jackson, Inger, Parnell, & Bearhop, 2011). Changes in isotopic values and niche widths over time can reveal changes in diet for an animal if prey isotopic values are distinct (Hobson, Schell, Renouf, & Noseworthy, 1996), which may be due to shifts in foraging behavior or changes in the abundance and distribution of prey.

**Figure 1:**
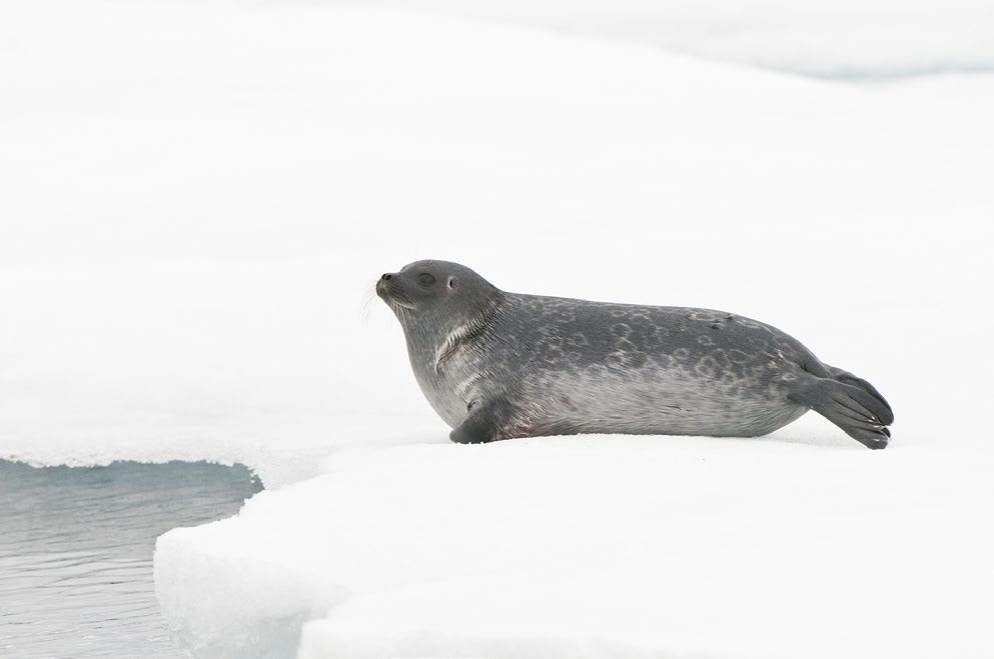
Photograph of a ringed seal resting on the sea ice. Photograph courtesy of Rinie van Meurs

Marine predators, such as ringed seals (*Pusa hispida*; Figure 1), play a role as indicator species, and their stable isotope values can offer insights into shifts in ecological communities (Harwood, Smith, George, et al., 2015; Yurkowski, Hussey, Ferguson, & Fisk, 2018). Ringed seals are a circumpolar, ice‐dependent species (Reeves, 1998), found in both pack and shorefast ice which is used by the seals for reproduction, molting, and hauling‐out (McLaren, 1958; Smith & Stirling, 1975). While ringed seals show high site fidelity in the winter and spring, they will range extensively in the late summer after sea ice break‐up (Kelly et al., 2010), which may expose them to a variety of prey items. As opportunists, ringed seals feed on over 70 species across the Arctic, including fish (e.g., Arctic cod, *Boreogadus saida*; capelin, *Mallotus villosus*) and crustaceans (e.g., amphipods, euphausiids; Dehn et al., 2007; Lowry, Frost, & Burns, 1978, 1980; McLaren, 1958). As ringed seals are generalists, shifts in prey availability will be reflected in their diets, and these variations in diet may be archived in stable isotopes of metabolically inactive tissues such as claws (Carroll et al., 2013; Ferreira, Loseto, & Ferguson, 2011).

Ringed seal claws can represent a stable isotope diet record up to approximately a decade, limited by claw wear at the tip from the creation and maintenance of breathing holes (McLaren, 1958; Smith & Stirling, 1975). Ringed seal claws have growth‐layer groups (GLGs)—alternating light and dark annuli which are likely demarcated by molting, or fasting and foraging cycles (Ferreira et al., 2011). Approximately one year of claw growth is represented by each pair of light and dark annuli, and therefore can be used to estimate the minimum age of the seal (Benjaminsen, 1973; McLaren, 1958). Light annuli are likely formed during the hyperphagic summer period, from May to winter (October to January), while dark annuli are formed during the period of reduced dietary intake (Ferreira et al., 2011).

Ringed seal diets may reflect changes in abundance and distribution of their prey (Provencher, Gaston, O'Hara, & Gilchrist, 2012; Rose, 2005) associated with changing sea ice dynamics and warming temperatures (Lindsay & Zhang, 2005; Proshutinsky, Dukhovskoy, Timmermans, Krishfield, & Bamber, 2015; Rigor, Wallace, & Colony, 2002; Stroeve et al., 2011). Loss of sea ice has been associated with changing percent frequency of prey occurrence in ringed seal stomach contents, with a 27.0% occurrence decrease in invertebrates, and increases in fish (e.g., 27.2% for Arctic cod; 45.8% for rainbow smelt, *Osmerus mordax*) in the Bering Strait (Crawford, Quakenbush, & Citta, 2015). Within Amundsen Gulf and Cumberland Sound, ringed seal niche widths have increased in recent years (1990–1996) compared to historical periods (1999–2011), which may be due to subarctic fish (e.g., capelin and *Ammodytes* spp.) shifting their range northwards due to rising ocean temperatures (Rose, 2005; Young & Ferguson, 2014; Yurkowski, Ferguson, Semeniuk, et al., 2016). In the Hudson Bay, high δ^15^N was linked to air temperatures between −5°C and −2°C in the spring, which was hypothesized to be due to increased feeding upon capelin (Young & Ferguson, 2014). Further, warming of the oceans may result in a shift to a pelagic‐dominated system, due to reduced sea ice facilitating zooplankton grazing on phytoplankton blooms, which in turn reduces the export of energy from primary production to the benthic community (Bluhm & Gradinger, 2008; Grebmeier, Cooper, Feder, & Sirenko, 2006). This shift to an ecosystem dominated by pelagic species is supported by stable isotope evidence that suggests that ringed seals feed on more pelagic organisms during years with less sea ice (Carroll et al., 2013). While the impact of changing environmental conditions on stable isotopes has been assessed in regions such as the Hudson Bay (e.g., Young & Ferguson, 2014), studies on variation in stable isotopes in ringed seals of the Beaufort Sea are limited. Therefore, the objective of our study is to examine interannual and spatial trends in δ^13^C and δ^15^N of ringed seals based on claw annuli collected from 1974 to 2011 in the Beaufort Sea. As ringed seals are opportunistic feeders, we expected that changes in prey availability driven by environmental variability would be reflected within seal diets and subsequently, their stable isotope values.

We tested our hypothesis by comparing ringed seal claw stable isotopes from seals killed by polar bears (*Ursus maritimus*) collected in the Beaufort Sea from 1974 to 2011 to environmental conditions that may facilitate shifts in community composition, including climate patterns, sea ice dynamics, and temperature. Additionally, we expected that seal stable isotopes would be influenced by body condition and productivity, as well as geographic location of the site where the seal was sampled. Body condition and productivity can indicate prey availability, which is influenced by sea ice conditions (Harwood, Smith, & Melling, 2000; Harwood, Smith, Melling, Alikamik, & Kingsley, 2012). Stable isotopes may be influenced by geographic distribution of individuals, due to spatial patterns in stable isotopes (e.g., decreasing δ^13^C eastward in the Beaufort Sea; Dunton, Schonberg, & Cooper, 2012; Schell, Saupe, & Haubenstock, 1989). Lastly, we hypothesized that borealization of fish communities in the Arctic would result in a larger range of prey available for seal consumption, resulting in a trend of larger niche widths over time. We did not attempt to infer ringed seal diet contributions because of the numerous possible prey sources within the ringed seal diet and a lack of prey stable isotope data that match our ringed seal data both spatially and temporally.

## 2 METHODS

### 2.1 Study area

**Figure 2:**
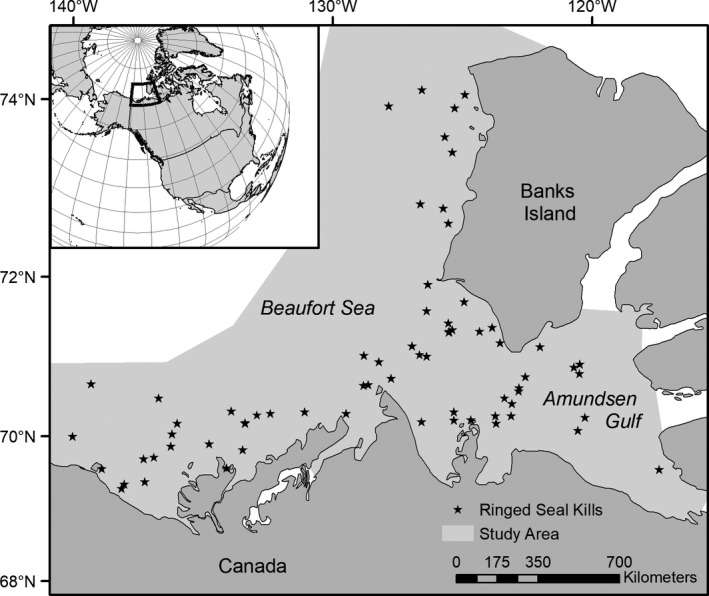
Map of the Beaufort Sea, showing locations of ringed seals (*n* = 66) killed by polar bears and collected between 1974 and 2011. Ringed seal kills (*n* = 24) without known geographic coordinates are not shown. Light gray indicates area where environmental variables were extracted for the study

The study area consists of the Beaufort Sea and Amundsen Gulf, north of the Yukon and Northwest Territories, Canada (Figure 2). This region is almost completely covered by pack and land‐fast sea ice in winter, and the open water season generally lasts from June through September (Galley, Else, Howell, Lukovich, & Barber, 2012; Galley, Key, Barber, Hwang, & Ehn, 2008). Within the Beaufort Sea, increasing easterly winds and sea surface temperatures have resulted in loss of sea ice and changes to sea ice dynamics (Wood et al., 2013). These changes include earlier break‐ups, delayed freeze‐ups, thinner sea ice cover, and reduced multi‐year sea ice concentration (Frey, Moore, Cooper, & Grebmeier, 2015; Galley et al., 2008, 2016). From 1979 to 2013, there were trends of earlier sea ice melt onset (−2.7 days/decade) and later freeze onset (6.4 days/decade), resulting in the open water season increasing about 9 days/decade (Stroeve et al., 2014). Further, sea ice concentration (Junuary–October) has decreased from 4.3% to 9.3% concentration/decade in the Beaufort Sea (Stern & Laidre, 2016).

The Beaufort Sea consists of several important features, including the Cape Bathurst Polynya and the Beaufort Gyre. The Cape Bathurst Polynya, a large stretch of open water, forms early in the year and is an important source of biological productivity within the area (Arrigo & van Dijken, 2004). The Beaufort Gyre is a wind‐driven ocean circulation feature within the Beaufort Sea and moves large quantities of sea ice and freshwater through the region (Proshutinsky, Bourke, & McLaughlin, 2002). Ocean heat content has almost doubled within the Beaufort Gyre between 1987 to 2017, which contributes to sea ice loss within the Beaufort Sea (Timmermans, Toole, & Krishfield, 2018).

### 2.2 Sample collection

Ringed seal claws were opportunistically collected from polar bear kill sites within 150 km of shore while conducting polar bear monitoring studies from 1974 to 2011, from April 3 to June 6 (Figure 2). In total, 93 ringed seal (23 female, 26 male, and 44 unknown) claws were collected (April: *n* = 52; May: *n* = 34; June: *n* = 7). Front flippers and jaws were collected from the kill sites, if available, for age and sex determination. Samples were frozen at −20°C until analysis. Observations of age class and sex of the seal were recorded if possible. Age of the seal was obtained or verified using canine teeth and GLGs on claws (McLaren, 1958; Stewart, Stewart, Stirling, & Street, 1996). We followed Stewart et al. (1996) to decalcify and age teeth (*n* = 46). Species and sex of the samples were verified using DNA analysis by Wildlife Genetics International (Nelson, British Columbia). All sampling was approved by the University of Alberta Biosciences Animal Care and Use Committee, the Government of the Northwest Territories, and the local Hunters and Trappers Committees, in accordance with guidelines from the Canadian Council on Animal Care.

One digit was cut from the front flippers, and excess tissue was removed using a scalpel. We primarily extracted digit no. I; however, if this digit was not available, we used digit no. II. No differences in stable isotopes have been found between digits for corresponding annuli (Ferreira et al., 2011). Claws were soaked in water until the dermis and cuticle skin softened and loosened from the unguis. Claws are made of keratin and therefore are lipid‐poor tissues (Newsome, Clementz, & Koch, 2010). However, to prevent ^13^C depletion due to lipids following Carroll et al. (2013), claws were cleaned using a 2:1 chloroform:methanol solution and rinsed with distilled water. For each claw, the number of dark and light annuli was recorded, and the age of the claw estimated using claw GLGs by two observers. The year in which each claw annulus was grown was determined by subtracting the annulus position along the claw (the number of annuli from the base of the claw) from the year of collection from the kill site. Claws were sectioned by light annuli using a rotary tool, set to 14,000 rev/min, that powdered the claw. We did not sample from pups, as their claw material would reflect maternal input from nursing.

### 2.3 Stable isotope analysis

All nitrogen and carbon stable isotope analyses were completed by the Great Lakes Institute for Environmental Research facility at the University of Windsor, using a Thermo Delta V Advantage Mass spectrometer with a Costech 4010 Elemental Combustion system and a ConFlo IV gas interface. The delta (*δ*) notation, measured in parts per thousand (‰), is used to express the stable isotope ratios:

(1) $$\delta X\left(‰\right)=\frac{R_{\text{sample}}-R_{\text{standard}}}{R_{\text{standard}}}\times 1,000$$

where *X* is the heavy isotope of the element (^15^N or ^13^C), and *R* is the ratio of heavy to light isotopes for nitrogen (^15^N/^14^N) or carbon (^13^C/^12^C) for the ringed seal claw sample (*R*_sample_) or the standard (*R*_standard_; δ^13^C values—Pee Dee Belemnite, δ^15^N—atmospheric nitrogen). Precision was determined based on standard deviations of replicates, using the following standards (*n* = 95): bovine liver (NIST1577c), tilapia muscle, USGS 40, and urea. Precision was ≤0.19‰ for δ^15^N and ≤0.15‰ for δ^13^C. The standard deviation of replicate samples was 0.08‰.

Before analysis, we corrected all ringed seal δ^13^C values for the Suess effect: the increasing amounts of ^13^C‐depleted anthropogenic CO_2_ causing a depletion in δ^13^C in dissolved inorganic carbon within oceans (Keeling, 1979; Revelle & Suess, 1957). The Suess effect correction has previously been applied to ringed seal stable isotopes (Carroll et al., 2013). The Suess effect correction factor was applied to the δ^13^C of all seal claw samples, using the following formula (Misarti, Finney, Maschner, & Wooller, 2009):

(2) $$\text{Suess}\;\text{effect}\;\text{correction}\;\text{factor}=a^{b\times 0.027}$$

where the variable *a* is the maximum annual rate of decrease in δ^13^C, which is −0.014 in the North Pacific (Quay, Tilbrook, & Wong, 1992); the variable *b* is the year in which the claw annulus was grown, minus 1,850 (i.e., start of the Industrial Revolution).

### 2.4 Statistical analyses

We used R version 3.6.1. for all statistical analyses (R Development Core Team, 2019). Ringed seal stable isotopes were compared between individuals of known sex and age classes using one‐way ANOVAs to determine whether the populations could be pooled. Unless a natal tip is present, claw annuli count only provide the age of the claw, not the individual's age. Therefore, only isotopes of the newest claw annulus were compared between age classes, to avoid incorrect assignment of a seal's older annuli to an age class. Shapiro–Wilk tests and Levene's tests were used to assess normality and homogeneity of variances respectively, for both carbon and nitrogen isotopes.

Linear mixed‐effects models, accounting for temporal pseudoreplication within an individual by using a nested structure of year and seal ID random effects (Crawley, 2012), were used to assess patterns in the claw annuli stable isotopes, with either δ^13^C or δ^15^N as the response variable (Crawley, 2012). Stepwise model selection was completed using Akaike's information criterion for small sample sizes (AIC_c_) to determine a top model. All biologically relevant interactions between variables were tested. When AIC_c_ < 2, the most parsimonious model was selected. Before model selection, we assessed multicollinearity between explanatory variables using Pearson correlation coefficients and excluded factors with coefficients > 0.7. We also used the variance inflation factor (VIF) to check for multicollinearity, using a cutoff of VIF > 10 (no VIFs exceeded 2.36). Durbin–Watson tests were used to assess each model's residuals for temporal autocorrelation.

### 2.5 Models and variables

#### 2.5.1 Climate index model

We first created a climate index model on the full data set, which included samples from all years in the study (1964–2011). The climate index model included the Pacific Decadal Oscillation (PDO), the Arctic Oscillation (AO), and the Arctic Ocean Oscillation (AOO), all of which influence sea ice dynamics (Lindsay & Zhang, 2005; Proshutinsky et al., 2015; Rigor et al., 2002; Stroeve et al., 2011). The PDO index describes sea surface temperature anomalies, with warm and cool phases (Mantua & Hare, 2002). The AO describes sea level pressure anomalies, and has positive and negative phases (Rigor et al., 2002). During the positive phase, the polar vortex strengthens, retaining cold air within the Arctic and influencing sea ice thickness and sea ice loss (Liu, Curry, & Hu, 2004; Rigor et al., 2002). The AOO consists of cyclonic and anticyclonic circulation regimes (Proshutinsky & Johnson, 1997). Within the Beaufort Sea, cyclonic circulation regimes of the AOO consist of cyclonic sea ice drift, warm and humid temperatures, increased ice melt, and increased sea ice and freshwater export (Proshutinsky et al., 2002; Proshutinsky & Johnson, 1997; Proshutinsky, Polyakov, & Johnson, 1999). Typically, cyclonic and anticyclonic circulations alternate every 5–7 years (Proshutinsky & Johnson, 1997). However, as of 1997, circulation has persisted in an anticyclonic regime (Proshutinsky et al., 2015). Data for the PDO and AO indices were obtained from NOAA (Boulder, Colorado, USA; PDO: https://www.ncdc.noaa.gov/teleconnections/pdo/, AO: https://www.cpc.ncep.noaa.gov/products/precip/CWlink/daily_ao_index/ao.shtml). We obtained data on the AOO from the Woods Hole Oceanographic Institution (Woods Hole, MA, USA; https://www.whoi.edu/page.do?pid=66578). As the Beaufort Sea is highly seasonal, we provided both annual and seasonal means for both the PDO and AO. Seasons were classified as follows: spring (April–June), summer (July–September), Autumn (October–December), and winter (January–March). Before inclusion in the candidate model, we assessed which PDO and AO temporal period to include based on minimizing AIC_c_ values. We also included lag variables (the previous annual period) for PDO, AO, and AOO.

#### 2.5.2 Environmental model

The climate index model was followed by an environmental model which included sea ice, sea surface temperature (SST), and air temperature variables, and carried forward significant variables from the climate index model. The model was restricted in time from 1982 to 2011, which is when satellite data on sea ice concentration and sea surface temperature was available. Data from 1986 were excluded due to lack of air temperature data from August to November. We did not include lag variables, to avoid dropping additional samples due to the temporal restriction in available satellite data. We determined annual sea ice break‐up and freeze‐up dates, as well as open water duration, using SSM/I satellite sea ice concentration data (National Snow and Ice Data Center, Boulder, CO, USA; http://nsidc.org/), clipped to the study area (Figure 2). Daily mean sea ice concentrations were calculated to determine break‐up and freeze‐up dates, based on a threshold of 50%. We chose a 50% threshold to be comparable to polar bear studies, which commonly use this percentage (Stern & Laidre, 2016). The break‐up date was defined as the first ordinal date in which sea ice concentration was < 50%, while the freeze‐up date was the first ordinal date in which sea ice concentration remained above that threshold. Open water duration was the difference between the break‐up and freeze‐up dates. In addition, we determined the sea surface and air temperatures for the Beaufort Sea. SSTs were obtained from the NOAA optimum interpolation SST v2 data set (NOAA/OAR/ESRL PSD, Boulder, Colorado, USA; https://www.esrl.noaa.gov/psd/) and resampled to match the SSM/I sea ice concentration resolution. The mean SSTs for summer were determined for the study area. Mean air temperatures for each season were calculated from the Environment and Climate Change Canada historical climate data set (http://climate.weather.gc.ca/). We used two climate stations (Sachs Harbour Climate: 71.99°N, 125.25°W; Sachs Harbour A: 71.99°N, 125.24°W), as not all years had data at each station. To determine whether the data from both stations could be pooled, we used a paired *t* test and compared overlapping years.

#### 2.5.3 Biological model

We compared ringed seal body condition and productivity to stable isotope values in a biological model that was restricted from 1992 to 2006 due to limited availability of ringed seal biological measures within the literature. We could not use body condition data from our sampled seals as our samples were obtained from polar bear kills, in which often most of the seal's fat was consumed and remaining tissue was limited. A measure of the population's body condition, based on a length‐mass‐blubber depth index of adult female ringed seals, was included from Harwood, Smith, Melling, et al. (2012). We included body condition in the model as either low (below overall median) or high (above overall median), with the assumption that the sampled seals reflect the overall population's health within the Beaufort Sea. We included annual ovulation rates of adult female ringed seals and annual percentage of ringed seal pups in the harvest as measures of productivity from Harwood, Smith, Melling, et al. (2012). We included a proportional width index (PWI), which is a measure of ringed seal teeth growth layers, from Nguyen et al. (2017). PWIs are correlated with ringed seal productivity (Nguyen et al., 2017) and may represent a measure of somatic growth (Wittmann et al., 2016). Additionally, we included a lagged PWI variable from the previous annual period, to assess carryover effects. Significant variables from the climate index and environmental models were included in the biological model.

#### 2.5.4 Location model

To assess the effects of geographic location of the kill site, δ^13^C and δ^15^N of the newest light annuli were compared to the ringed seal kill site's longitude, latitude, bathymetry, and distance from shore using a linear mixed‐effects regression. Ringed seals show interannual site fidelity during the spring and early summer, ranging only up to 2 km^2^, and may remain in the same home range area for up to 10 months each year (Kelly et al., 2010). As the samples were collected in April and May, we assumed that the location of the kill site would approximately reflect the area that the ringed seal most commonly used during the formation of the light annuli. However, during ice‐free periods (approximately 2–4 months), ringed seals may forage across significant distances (>100 km; Harwood, Smith, Auld, Melling, & Yurkowski, 2015; Kelly et al., 2010). As such, a caveat to this analysis is that 2–4 months of the May to January annuli growth period may include the seal traveling extensively to forage, which may not accurately reflect the sampling location.

As the dates of samples with geographic locations did not completely overlap the sampling periods for the climate, environmental, or biological models, we analyzed the location data without carrying over terms from the previous models. The distance to shore was measured from the kill site to the closest coastline. Bathymetry at the kill site location, which may influence prey available to the seal, was determined using the International Bathymetric Chart of the Arctic Ocean (IBCAO) version 3.0 (Jakobsson et al., 2012).

### 2.6 Niche width

To determine the niche width of ringed seals, we used SIBER (Stable Isotope Bayesian Ellipses in R) version 2.1.3 (Jackson et al., 2011). SIBER requires at least three samples per group for to generate ellipses, and smaller sample sizes result in higher uncertainty (Jackson et al., 2011). Therefore, we chose to assess niche width using decades, rather than years, to improve model estimates. We compared ringed seal stable isotopes between the 1960s, 1970s, 1980s, 1990s, and 2000s. We did not include the 2010s as it had less than three samples. Claw annuli are often not grown within the same decade, and therefore, a mean value cannot always be associated with a specific decade of growth. Variation in stable isotopes is expected to be higher between individuals than within an individual, and pseudoreplication is not accounted for within SIBER. Therefore, we randomly selected one annulus from each ringed seal claw to represent that individual's stable isotopes and grouped the samples into decades based on the year that claw annulus was grown. Niche width is represented by the Bayesian estimate of the standard ellipse area (SEA_b_). Bayesian inference was used to compare ringed seal niche widths (SEA_b_) between decades, by comparing the proportion of posterior estimates that were higher in one decade to another, in relation to the total posterior estimates.

## 3 RESULTS

**Figure 3:**
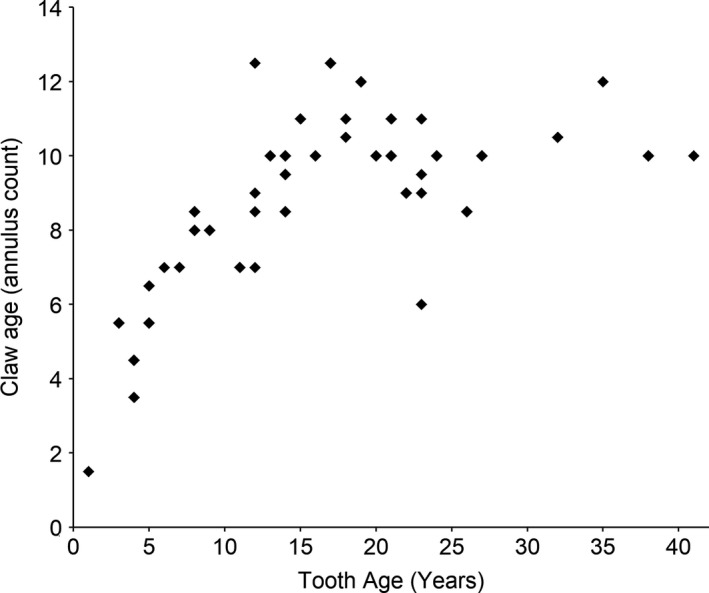
Comparison of tooth age (years) and claw age (annulus count) of Beaufort Sea ringed seals (*n* = 46)

**Figure 4:**
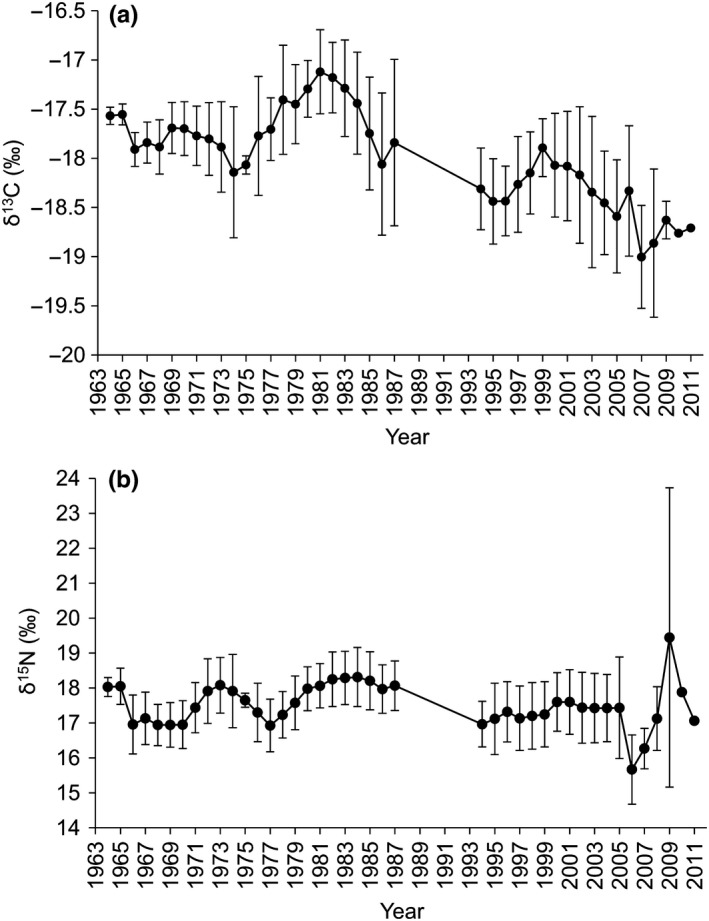
Claw stable isotope values of (a) δ^13^C and (b) δ^15^N from Beaufort Sea ringed seal claw annuli (*n* = 93 seals; 714 claw annuli) collected between 1974 and 2011, and grown between 1964 and 2011

Most samples were taken from adult seals (25 juveniles, 68 adults) based on claw and tooth age (Figure 3). The maximum number of light annuli counted on a ringed seal claw was 12, with a mean claw age of 7.8 ± 0.3 years. The oldest seal in the study, based on tooth age, was 41 years (claw age: 10 years). In total, 714 claw annuli were analyzed for stable isotopes, with years of growth ranging from 1964 to 2011 (Figure 4). No difference in carbon and nitrogen stable isotopes was found between known sexes or age classes (*p* > .05); therefore, samples were pooled for analyses. When the population was pooled, the mean Suess‐corrected δ^13^C was −17.9 ± 0.6‰ (range: −20.15 to −15.63‰), while δ^15^N was 17.6 ± 1.0‰ (range: 14.57 to 20.24‰).

The climate model included 93 ringed seals (714 claw annuli). The top δ^13^C climate model only included AOO (Tables 1, 2, 3, 4), with δ^13^C increasing with cyclonic circulation regimes. AIC_c_ indicated that the best fitting δ^15^N climate model was the null.

**Table 1:** Selection of seasonal (Annual, Winter: January–March, Spring: April–June, Summer: July–September, Autumn: October–December) climate index and air temperature metrics for inclusion into climate and environmental models, respectively, with a response variable of either δ^13^C or δ^15^N of ringed seal claw annuli from 1964 to 2011 in the Beaufort Sea

Response variable	Season	AIC_c_		
		Pacific decadal oscillation	Arctic oscillation	Air temperature
δ^13^C	Annual	764.0	654.8	488.7
	Winter	765.6	655.7	488.8
	Spring	767.0	654.4	**487.8**
	Summer	766.1	**652.4**	489.4
	Autumn	**762.0**	656.3	488.8
δ^15^N	Annual	1,084.7	967.0	601.1
	Winter	1,087.4	968.7	601.4
	Spring	1,088.3	967.6	601.4
	Summer	1,085.8	**964.0**	**600.1**
	Autumn	**1,083.1**	969.1	601.3

Note

The seasonal metric that minimized AIC_c_ (bolded) was used within the candidate climate index and environmental models.

**Table 2:** Selection of correlated environmental metrics, based on minimizing AIC_c_, modeled against δ^13^C or δ^15^N of ringed seal claw annuli from 1964 to 2011 in the Beaufort Sea

Response variable	Sea ice metric	AIC_c_
δ^13^C	50% BREAK	491.4
	50% FREEZE	492.7
	OPEN WATER	493.6
	SUMMER SST	**486.5**
δ^15^N	50% BREAK	597.9
	50% FREEZE	600.7
	OPEN WATER	597.0
	SUMMER SST	**595.3**

Note

Top terms included within environmental model are bolded. 50% BREAK = first day sea ice concentration < 50%, 50% FREEZE = first day sea ice concentration remains > 50%, OPEN WATER = duration between 50% BREAK and 50% FREEZE, SUMMER SST = summer (July–September) sea surface temperature.

**Table 3:** Top five climate (1964–2011), environmental (1982–2011 except 1986 due to missing data), biological (1992–2006), and geographic location models based on AIC_c_ scores for δ^13^C and δ^15^N of ringed seal claw annuli

Model	Response	Rank	Model	AIC_c_	ΔAIC_c_	AICc Wt
Climate	δ^13^C	1	AOO	751.5	0.0	0.60
		2	LAGGED AOO	753.7	2.3	0.10
		3	AUTUMN PDO + AOO	754.8	3.3	0.11
		4	AUTUMN PDO + LAGGED AO + AOO	757.0	5.6	0.037
		5	AUTUMN PDO + LAGGED AOO	757.4	6.0	0.030
	δ^15^N	1	NULL	1,081.3	0.0	0.50
		2	SUMMER AO	1,083.5	2.2	0.17
		3	AUTUMN PDO	1,083.9	2.7	0.13
		4	LAGGED AO	1,084.7	3.4	0.089
		5	AUTUMN PDO + SUMMER AO	1,085.4	4.1	0.063
Environmental	δ^13^C	1	AOO	483.25	0.00	0.48
		2	NULL	485.43	2.18	0.26
		3	SUMMER SST + AOO	486.81	3.56	0.081
		4	SPRING AT + AOO	487.81	4.56	0.049
		5	SUMMER SST	488.08	4.83	0.043
	δ^15^N	1	SUMMER SST	595.25	0.00	0.81
		2	SUMMER AT	600.08	4.83	0.073
		3	NULL	600.73	5.48	0.053
		4	SUMMER SST:SUMMER AT	601.27	6.02	0.040
		5	SUMMER SST + SUMMER AT	602.62	7.37	0.020
Biological	δ^13^C	1	LAGGED PWI + AOO	334.1	0.0	0.65
		2	LAGGED PWI + OVULATION +AOO	336.5	2.4	0.20
		3	LAGGED PWI + CONDITION +AOO	338.8	4.7	0.063
		4	LAGGED PWI + %PUPS + CONDITION +AOO	340.5	6.4	0.027
		5	LAGGED PWI + %PUPS + CONDITION + %PUPS:CONDITION + AOO	341.0	6.8	0.021
	δ^15^N	1	% PUPS + SUMMER SST	402.58	0.00	0.58
		2	SUMMER SST	405.50	2.92	0.14
		3	% PUPS + OVULATION +SUMMER SST	406.27	3.70	0.092
		4	% PUPS + PWI +CONDITION + SUMMER SST	406.35	3.78	0.088
		5	OVULATION + SUMMER SST	408.05	5.48	0.038
Location	δ^13^C	1	LONGITUDE	141.57	0.00	0.92
		2	LATITUDE + LONGITUDE	146.43	4.86	0.081
		3	LONGITUDE + SHORE	153.58	12.01	0.0020
		4	LATITUDE + SHORE	153.78	12.21	0.0020
		5	LATITUDE + LONGITUDE +SHORE	158.16	16.59	<0.00
	δ^15^N	1	SHORE + LONGITUDE	205.10	0.00	0.99
		2	SHORE + BATHY	216.23	11.13	0.004
		3	BATHY + LONGITUDE +LATITUDE	217.11	12.01	0.002
		4	SHORE + BATHY +LONGITUDE	220.38	15.27	<0.00
		5	SHORE + BATHY +LATITUDE	220.88	15.78	<0.00

Note

“:” denotes interaction.

Abbreviations: % PUPS, percentage of pups in harvest; AO, Arctic Oscillation; AOO, Arctic Ocean Oscillation; AT, air temperature; BATHY, bathymetry (m); CONDITION, body condition; OVULATION, ovulation rate; PDO, Pacific Decadal Oscillation; PWI, proportional width index; SHORE, distance to shore (km); SST, sea surface temperature.

**Table 4:** The estimate, standard error (*SE*), *t*‐value, and *p*‐value for variables of the top climate (1964–2011), environmental (1982–2011 except 1986 due to missing data), and biological (1992–2006) models influencing Suess‐corrected carbon stable isotopes of ringed seal claw annuli in the Beaufort Sea

Model	Coefficient	Estimate	*SE*	*t*‐Value	*p*‐Value
Climate	Intercept	−18.16	0.08	−223.93	<.001
	AOO (CCR)	0.39	0.11	3.70	.0012
Environmental	Intercept	−18.29	0.12	−152.20	<.001
	AOO (CCR)	0.43	0.20	2.18	.044
Biological	Intercept	−22.66	0.95	−23.89	<.001
	LAG PWI	15.08	3.24	4.65	.0015
	AOO (CCR)	−0.042	0.11	−0.40	.70

The environmental model included 74 seals (474 claw annuli). Air temperatures were not significantly different between the two climate stations (*t*_(13)_ = −1.60, *p* = .13) so we pooled data from both stations. The top δ^13^C environmental model supported by AIC_c_ included only AOO (Tables 2, 3, 4). The top δ^15^N environmental model included only summer SST, with increasing δ^15^N at lower summer SSTs (Table 5).

**Table 5:** The estimate, standard error (*SE*), *t*‐value, and *p*‐value for variables of the top environmental (1982–2011 except 1986 due to missing data) and biological (1992–2006) models influencing nitrogen stable isotopes of ringed seal claw annuli in the Beaufort Sea from 1964 to 2011

Model	Coefficient	Estimate	*SE*	*t*‐Value	*p*‐Value
Environmental	Intercept	17.90	0.15	129.31	<.001
	Summer SST	−0.11	0.043	−2.66	.025
Biological	Intercept	17.58	0.16	112.14	<.001
	%Pups	0.39	0.13	2.99	.014
	Summer SST	−0.19	0.036	−5.24	.00030

Note

The climate model estimates are not presented, as the top model was the null model.

The biological model included 39 seals (337 claw annuli). Only AOO and lagged PWI remained in the top δ^13^C biological model (Tables 3 and 4). AOO did not significantly influence the δ^13^C biological model (Table 4); however, it was left in the model due to its previous significance in the models. δ^13^C increased with an increasing PWI. The top δ^15^N biological model included percentage of pups in the harvest, and summer SST (Tables 3 and 5). δ^15^N increased with higher percentages of pups in the harvest.

The geographic location model included 66 seals. Longitude of the ringed seal kill site was related to δ^15^N and weakly to δ^13^C (Table 6). Eastward longitudes had decreasing δ^13^C and increasing δ^15^N. As well, δ^15^N was related to the distance of the kill site from shore. Ringed seals killed further offshore had higher δ^15^N than nearshore seals. Bathymetry was not related to either δ^13^C or δ^15^N.

**Table 6:** The estimate, standard error (*SE*), *t*‐value, and *p*‐value for variables in the models examining the effect of location on carbon and nitrogen stable isotopes of ringed seal claw annuli in the Beaufort Sea

Model	Coefficient	Estimate	*SE*	*t*‐Value	*p*‐Value
δ^13^C	Intercept	−21.86	1.69	−12.96	<.001
	Longitude	−0.026	0.013	−2.01	.05
δ^15^N	Intercept	22.57	2.64	8.55	<.001
	Distance from Shore (km)	0.0011	0.0046	2.45	.017
	Longitude	0.044	0.020	2.13	.037

**Figure 5:**
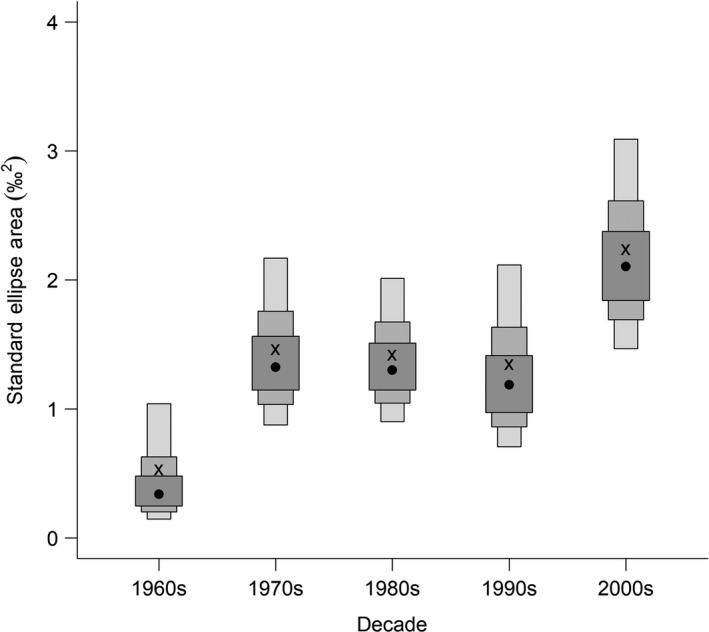
Density plot produced by SIBER of the Bayesian estimate of the standard ellipse area (SEA_b_; ‰^2^) for Beaufort Sea ringed seal claw annuli δ^13^C and δ^15^N stable isotope values. The mode for each decade is represented by the black circle, and credible intervals of 50%, 75%, and 95% are represented by the shaded boxes

SEA_b_ varied between decades for ringed seals in the Beaufort Sea (Figure 5). Compared to all other decades, niche width was smallest in the 1960s (97% to 99%) and largest in the 2000s (94% to 99%). Between the 1970s and 1990s, SEA_b_ remained relatively consistent (38% to 62%). The niche widths of the 2000s and 1960s did not overlap; all other decades overlapped (Figure 5).

## 4 DISCUSSION

Ecosystem changes in response to shifting environmental conditions within the Arctic may be captured in stable isotopes of ringed seals, as demonstrated in our study. Our sampled seal claws captured up to 12 years of dietary history for an individual, which demonstrates the value in sampling metabolically inactive tissues and allows us to examine the influence of shifting environmental conditions upon ringed seal stable isotopes up to a decade before sampling. We found that the AOO, summer SST, seal productivity, and kill site location were related to claw stable isotopes, showing that a variety of factors affect variation in ringed seal diet histories. Further, we found that niche width of seals had increased from the 1960s to 2000s, which may correspond to range expansions of subarctic prey species. However, our results highlight the complexity of inferring ecosystem shifts from isotopic values, and interpretations from isotopic values alone should be made cautiously.

Our study had several limitations, including lack of dietary information, baseline isotopic data, and time lags, that may influence our interpretations. First, because our samples were opportunistically taken from polar bear killed seals, information on seal stomach contents was not available. Bayesian mixing models, which use stable isotopes to infer dietary contributions, could be an alternative to stomach content analyses. However, we chose not to run Bayesian mixing models for diet estimation because: (a) ringed seals consume numerous prey sources leading to underdetermined models and a lack of unique dietary solutions and (b) prey source stable isotope values were unavailable for our study area and/or period. However, as a result, we are unable to determine how diet contributions changed with environmental conditions and can only make inferences. Further, we do not have annual baseline isotopic data for the Beaufort Sea. This data could be obtained through stable isotopes sampled at intervals from bowhead whale (*Balaena mysticetus*) baleen (Matthews & Ferguson, 2015; Pomerleau et al., 2018). However, without further data, we were unable to account for possible variation in isotopic baseline, which may be influenced by shifting environmental conditions (e.g., temperature, productivity; Casey & Post, 2011) that likely play a role in affecting ringed seal stable isotopes. Therefore, it is possible that changing isotopic baselines contribute to variation in δ^13^C and δ^15^N values. Lastly, while we accounted for climate index and PWI lags, we did not assess time lags in other environmental variables. It is possible that carryover effects occur within this system and future studies would benefit from further testing of lagged environmental variables.

The only climate index related to ringed seal claw stable isotopes was the AOO, with higher δ^13^C in cyclonic circulation regimes. Circulation regimes within the Arctic influence organic matter composition and primary producer dynamics (Pabi, Dijken, & Arrigo, 2008), which would affect primary producer δ^13^C and in turn, seal δ^13^C. Cyclonic circulation involves the release of low salinity water from the Beaufort Sea (Proshutinsky et al., 2002), which may increase δ^13^C of dissolved organic carbon due to higher salinity (Gillikin, Lorrain, Bouillon, Willenz, & Dehairs, 2006). As well, warmer waters and reduced sea ice in cyclonic regimes may facilitate phytoplankton blooms, in which rapid carbon fixation would lead to reduced ^13^C discrimination in primary producers and as a result, depleted ^13^C food resources for seals. One contradiction to this hypothesis is that, while δ^13^C of particulate organic matter increases with sea surface temperature (Goericke & Fry, 1994), we did not find that sea surface temperature or sea ice dynamics were related to ringed seal δ^13^C, although this pattern may be dependent on time lags. However, reduced sea ice thickness, which we did not examine within this study, may result in blooms of sympagic algae communities, as thinner sea ice allows more light penetration (Arrigo et al., 2012), resulting in enriched ^13^C through the food web (Arrigo & van Dijken, 2004; Goering, Alexander, & Haubenstock, 1990). As well, ice particulate organic matter and algae is enriched in ^13^C compared to suspended matter and phytoplankton, respectively (France, Loret, Mathews, & Springer, 1998; Gradinger, 2009). If seals are feeding on a more productive sympagic food web, it is possible that would result in their higher δ^13^C. Overall, increased uptake of enriched ^13^C by primary producers may result in elevated ^13^C in ringed seals.

Cyclonic circulation regimes may favor pelagic‐dominated communities (Bluhm & Gradinger, 2008; Proshutinsky et al., 1999, 2002; Proshutinsky & Johnson, 1997), which are characterized by depleted ^13^C (McConnaughey & McRoy, 1979). Conversely, our study found that ringed seals had higher δ^13^C in cyclonic years, for both current and lagged AOO. Carroll et al. (2013) attributed changes in stable isotopes within ringed seals and bearded seals (*Erignathus barbatus*) in the Chukchi and Bering Seas to be from the shift to a pelagic‐dominated community. The Beaufort Sea has lower benthic biomass than the Chukchi or Bering Seas (Dunton, Goodall, Schonberg, Grebmeier, & Maidment, 2005); therefore, the change to a pelagic‐dominated system may not be as drastic.

Warm temperatures promote a pelagic‐dominated food web (Bluhm & Gradinger, 2008; Grebmeier, Cooper, et al., 2006) and unlike δ^13^C, the relationship between δ^15^N and SST did support use of pelagic food webs. Pelagic organisms are often depleted in ^15^N relative to benthic due to a longer benthic food web (Iken, Bluhm, & Gradinger, 2005). However, benthic organisms are highly variable in δ^15^N (Iken et al., 2005). Overall, it is difficult to determine whether seals are feeding more on a pelagic food web without data on seal diet contributions, pelagic biomass, movement patterns, and/or isotopic baselines.

Alternatively, low δ^15^N in claw annuli with higher SSTs may be a result of seals feeding at a lower trophic level, changes to the isotopic baseline, or range expansions of subarctic prey species. Nitrogen is typically indicative of the individual's trophic level (Minagawa & Wada, 1984), suggesting that ringed seals may consume lower trophic level foods (e.g., invertebrates) at high SSTs. In warmer years, low trophic level prey species proliferate and are more abundant for consumption (Coyle & Pinchuk, 2003). However, without data on variation in the isotopic baseline, it is difficult to assess whether seals are feeding at lower trophic levels. It is likely that the isotope value at the base of the food web varies, as it is likely influenced by SST and productivity, among other factors (Casey & Post, 2011). Another possibility is that increased SSTs support the range expansion of subarctic fish into the Beaufort Sea, providing more abundant higher trophic level foods. For example, walleye pollock (*Theragra chalcogramma*) have extended their range into the Beaufort Sea since the 1970s (Frost & Lowry, 1983; Rand & Logerwell, 2011). Further, certain fish species within the Beaufort Sea (e.g., Arctic cod) show preference to colder waters (Logerwell, Rand, & Weingartner, 2011) or may have variable survivorship under different temperatures (Michaud, Fortier, Rowe, & Ramseier, 1996). For example, Arctic cod adults may be selected more by ringed seals during cold periods due to higher juvenile mortality (Gaden, Ferguson, Harwood, Melling, & Stern, 2009; Michaud et al., 1996). Therefore, higher trophic level prey may dominate ringed seal diets in cold years.

While environmental conditions influenced stable isotopes, sex and age were not related to an individual's stable isotope value indicating similar foraging habitats and prey between demographic groups. As a caveat within our study, not all individuals were of known sex and only the age class of the seal was assessed as claw annuli do not provide an exact age if the natal tip is missing. Polar bears may exhibit selection of seal age classes, with juveniles selected the least (Pilfold, Derocher, Stirling, Richardson, & Andriashek, 2012), which may bias our sample. Ringed seals have a highly variable diet (Dehn et al., 2007; Lowry, Frost, & Burns, 1978, 1980), and variation in foraging between demographic groups occurs based on stable isotopes (Dehn et al., 2005; Young & Ferguson, 2014; Young, Loseto, & Ferguson, 2010; Yurkowski, Ferguson, Semeniuk, et al., 2016) and stomach content analysis (Dehn et al., 2007; Lowry et al., 1980). Based on stomach contents, males consume more zooplankton and less fish than females, and fish consumption increases with age (Dehn et al., 2007; Lowry et al., 1980; Yurkowski, Ferguson, Semeniuk, et al., 2016). Further, resource partitioning may exist between demographic groups (Field, Bradshaw, Burton, Sumner, & Hindell, 2005; Newland, Field, Nichols, Bradshaw, & Hindell, 2009) but may not be detected by stable isotopes. For example, differences in foraging between sexes and ages detected by stomach content analyses were not reflected in stable isotopes within ringed seals of the Canadian Beaufort Sea (Dehn et al., 2007). As well, resource partitioning within ringed seals may vary by location (Yurkowski, Ferguson, Choy, et al., 2016; Yurkowski, Ferguson, Semeniuk, et al., 2016) and may not have occurred within our study area.

No support was found for different foraging strategies between demographic groups; however, stable isotope values were related to ringed seal population productivity, with δ^13^C increasing at high lagged PWIs and δ^15^N increasing with percentage of pups in the subsistence harvest. During fasting, δ^13^C decreases due to the use of ^13^C‐depleted lipids (Polischuk, Hobson, & Ramsay, 2001; Williams, Buck, Sears, & Kitaysky, 2007), and therefore, high δ^13^C may be related to increased body condition or growth in seals, which is indexed by PWI (Nguyen et al., 2017; Wittmann et al., 2016). Conversely, population body condition was not significantly related to δ^13^C or δ^15^N; however, the population body condition data from Harwood, Smith, Melling, et al. (2012) focused only on the Amundsen Gulf. It is possible that these body condition data do not well represent the Beaufort Sea ringed seals or the individuals sampled within our study. Ideally, measures of body condition would be taken from sampled individuals; however, sampled ringed seal carcasses were often mostly consumed and body fat measures were unavailable. Future studies on wild seals would benefit from comparisons between body condition and stable isotope values taken from captive animals.

Higher δ^15^N during years with high percentages of pups in the harvest could indicate foraging on more high trophic level species (e.g., fish) or nutritional stress. Ringed seal diets have shifted to include more high trophic level fish within the Bering and Chukchi Seas, which is linked to healthier and larger individuals (Crawford et al., 2015). Harwood, Smith, Melling, et al. (2012) found that ovulation rates increased with body condition, which may result in more harvested pups. Body condition was not a significant variable within our models, but we did not test for a lagged body condition variable, which may better reflect energy available for reproduction. However, elevated δ^15^N is also linked to nutritional stress because ^14^N is excreted (Hobson, Alisauskas, & Clark, 1993). In seals, lactation is energetically expensive and can elevate δ^15^N (Kurle & Worthy, 2001; Sinisalo, Jones, Helle, & Valtonen, 2007). Further, ringed seal pups are energetically expensive to raise (Lydersen, 1995). However, in our study, no difference in stable isotopes between sexes was found. In addition, Nguyen et al. (2017) found evidence for similar energy budgets between sexes based on PWIs. Together, this indicates reproduction likely was not increasing nutritional stress. However, demersal trawl surveys indicate higher fish abundance and northward range expansions of fish species in recent (2008) compared to historical (1977) surveys (Rand & Logerwell, 2011), and fish have increased in importance within diets of seals in the Bering and Chukchi Seas (Crawford et al., 2015). Therefore, we conclude that the elevated δ^15^N during years with a higher percentage of pups is due to ringed seals foraging at a higher trophic level and thus having more energy for reproduction, rather than nutritional stress.

Interpretations of diet may be influenced by the geographic location at which the individual forages. However, our analysis was a coarse comparison of the relationship between geographic location and stable isotopes values, due to the limitations imposed from sampling at a kill site and a lack of telemetry data. Ringed seals, particularly juveniles, may range long distances during the open water season and site fidelity may vary between age classes (Harwood, Smith, Auld, et al., 2015; Kelly et al., 2010). The period of extensive movement overlaps part of the light annuli growth period. We did not have tooth age data for all seals; however, polar bears primarily predated adult seals—which exhibit increased site fidelity—within this region (Harwood, Smith, Auld, et al., 2015; Kelly et al., 2010; Pilfold et al., 2012). As such, our results are preliminary and would be best reassessed with telemetry data collected through the season of annuli growth.

Individuals with kill sites located further east had depleted ^13^C and enriched ^15^N. The longitudinal pattern in ringed seal δ^13^C likely reflects the trend of δ^13^C depletion eastward from the Bering Sea to Beaufort Sea in zooplankton (Saupe, Schell, & Griffiths, 1989; Schell, Barnett, & Vinette, 1998). Previously, this trend was noted in migrating bowhead whales from the Bering to Beaufort Sea (Schell et al., 1989). As well, Dehn et al. (2007) found this trend of lower δ^13^C in ringed seals from Alaska to Canada. A trend in δ^15^N depletion eastward within the Beaufort Sea was found in zooplankton (Schell et al., 1998), but this was not reflected in ringed seal δ^15^N which increased eastward. It is possible that ringed seals are feeding at a higher trophic level in the eastern Beaufort Sea than further west.

Nitrogen sources within the food web may have resulted in reduced ringed seal δ^15^N close to shore. Nearshore organisms may have depleted ^15^N due to the source of nitrogen being terrestrially derived (Dunton et al., 2012). Coastal erosion and rivers (e.g., Mackenzie River) that empty into the Beaufort Sea are a source of terrestrially derived organic matter, which is then integrated into nearshore food webs (Naidu et al., 2000; Parsons et al., 1989). Fish inhabiting estuarine waters as compared to coastal waters of the Beaufort Sea were depleted in ^15^N (Dunton et al., 2012), and this depletion of δ^15^N may be reflected in ringed seals.

It is possible that seals shift their spatial distribution along these isotope gradients in response to variation in environmental factors (e.g., SST), resulting in the stable isotope patterns we observed. For example, increased δ^13^C during years of cyclonic AOO may be due to ringed seals shifting foraging locations westward, and lower δ^15^N with warming SST may be due to seals foraging further west or offshore. However, data on foraging locations of seals are unavailable.

Niche width increased from the 1960s to 2000s, indicating that ringed seal diet, or that of their prey, has diversified. A shift in community composition within Arctic ecosystems has occurred (Frost & Lowry, 1983; Jarvela & Thorsteinson, 1999; Rand & Logerwell, 2011), which may explain the increasing niche width. The number of macrobenthic species within the Bering Sea greatly exceeds the Beaufort Sea (Grebmeier, Cooper, et al., 2006; Sirenko, 2001), and some of these species may expand their ranges as northern waters warm and sea ice recedes. Some species, such as Bering flounder (*Hippoglossoides robustus*), have already expanded their distributions northward into the Beaufort Sea from the 1970s (Rand & Logerwell, 2011). As a result, the prey sources available to ringed seals may have increased over time. Ringed seals exhibit seasonal home range fidelity but may migrate between the Beaufort and Chukchi Seas (Harwood, Smith, & Auld, 2012; Kelly et al., 2010). Another possible explanation for the trend in increasing niche width is that ringed seals are migrating further toward the Chukchi Sea and therefore are being exposed to a larger number of prey species.

Overall, ringed seal stable isotopes are influenced by a variety of factors that must be considered when interpreting ecosystem dynamics. Age and sex of the individual were not related to stable isotope values. However, demographic groups, as well as geographic location and seal body condition, should be considered as possible confounding factors for diet interpretations in future studies. Further, future studies should investigate stable isotopes in relation to data on telemetry (e.g., dive data), prey populations, and additional time lags in environmental variables. While the Beaufort Sea ecosystem may be shifting toward a pelagic‐dominated community (Bluhm & Gradinger, 2008), our study found conflicting evidence—higher δ^13^C in cyclonic years and increasing δ^15^N at lower summer SSTs—for this shift. Warming temperatures and sea ice loss within the Arctic may result in changes to community composition and structure (Comeau, Li, Tremblay, Carmack, & Lovejoy, 2011; Galley et al., 2016; Post et al., 2013; Wood et al., 2013), and our study provides evidence that ringed seal diets have diversified since the 1960s in association with climate warming. Sea ice loss is expected to continue in the future (Gascard et al., 2017; Overland & Wang, 2013; Wang & Overland, 2009). Although the complexity of the ecosystem limits interpretation, continued monitoring of stable isotopes of ringed seals provides an archive of ecological changes within the Arctic food web.

## CONFLICT OF INTEREST

The authors declare that there is no conflict of interest.

## AUTHOR CONTRIBUTION

NPB, AED, and ESR: Experiment design. AED and ESR: Field sampling and data collection. NPB: Laboratory work and data analyses. NPB: Writing manuscript with input from AED and ESR.

## Data Availability

Data are archived and openly available on the University of Alberta Dataverse system (https://dataverse.library.ualberta.ca) at https://doi.org/10.7939/DVN/UTDOIR.
